# Genetic polymorphisms of 16 X-STR loci in the Hani population from Southwest China

**DOI:** 10.1080/20961790.2021.1877389

**Published:** 2021-06-16

**Authors:** Linlin Liu, Jinyong Yao, Yangzhi Huang, Lei Gao, Jiameng Dai, Xiaokun Yuan, Xiufeng Zhang, Shengjie Nie, Liping Hu

**Affiliations:** a School of Forensic Medicine, Kunming Medical University, Kunming, China; b Judicial Expertise Center, Kunming Medical University, Kunming, China; c Honghe Public Security Bureau, Honghe, China

**Keywords:** Forensic sciences, forensic genetics, forensic parameters, genetic polymorphisms, Hani population, X-STR, Yunnan Province

## Abstract

X chromosomal short tandem repeats (X-STRs) have the characteristics of both autosomal and uniparental genetic markers and have been shown to be particularly useful in forensic casework. However, relevant research or reports have not focused on X-STRs in the Hani population. To investigate the genetic variation and forensic efficiency of 16 X-STR loci in the Hani ethnic minority, we calculated the allele frequencies and forensic parameters of 451 (116 males and 335 females) unrelated healthy Hani individuals from Yunnan Province, Southwest China. All these loci are highly polymorphic in Hani individuals in Yunnan Province except DXS6800. The combined power of discrimination in males (PD_M_) and power of discrimination in females (PD_F_) were found to be 0.999 999 998 433 993 and 0.999 999 999 999 998, respectively. Furthermore, a population genetic structure investigation between the Yunnan Hani population and another 18 populations was performed using a principal component analysis, multidimensional scaling plot and neighbouring-joining phylogenetic tree and the findings illustrated that neighbouring populations and different nationalities in the same area appeared to have a closer evolutionary relationship. This study provides the first batch of X chromosome genetic polymorphism data of the Hani population in Yunnan Province, Southwest China and enriches the reference database of the Chinese minority population.

This is the first study of X-STR in the Hani population.

We calculated the allele frequencies and forensic parameters of 451 unrelated healthy Hani individuals from Yunnan Province, Southwest China.

All these loci are highly polymorphic in Hani individuals in Yunnan Province except DXS6800.

The genetic relationship between the Hani and other 18 nationalities was analyzed.

This study provides the first batch of X chromosome genetic polymorphism data of the Hani population in Yunnan Province, Southwest China and enriches the reference database of the Chinese minority population.

## Introduction

Autosomal short tandem repeats (STRs) are relatively established genetic markers that have been widely used in paternity testing and human identification, and they can effectively solve common forensic science problems [1, 2]. However, in some special cases, especially in complex kinship analyses, markers such as X and Y chromosomal STRs (X-STRs and Y-STRs, respectively) and mitochondrial DNA (mtDNA) are needed and complement each other with their unique genetic information [3–5]. X-STRs have the characteristics of both autosomal and uniparental genetic markers and have been proven to be particularly useful in certain cases, such as mother–son kinship tests, half-sister deficiency paternity tests, certain mixed strain types, paternity tests involving incest and next generation progeny relationship paternity tests could play an exclusionary role and help autosomal genetic marker testing lead to a positive conclusion [2, 6–8]. Therefore, X-STRs have been the focus of many studies in population genetics and forensic medicine [2, 9, 10]. Because the allele frequency distribution of X-STR loci is different in different populations, it is necessary to evaluate the allele frequency distribution of different populations.

The Sixth National Population Census of China in 2010 indicated that the population of the Hani ethnic minority in Yunnan Province is 1.63 million, which ranks second among ethnic minorities in Yunnan Province [11]. The Hani people in Yunnan Province are mainly distributed in Honghe Hani and Yi Autonomous Prefecture, Xishuangbanna Dai Autonomous Prefecture, Pu’er City and Yuxi City. The Hani ethnic group has had other historical names, although after 1949, “Hani” became the official name of the unified ethnic group according to the majority of the opinion of the people of this ethnic group. Hani belong to the Yi branch of the Sino-Tibetan language family. Before 1949, the Hani people did not have their own written language, and in some places, they used carved wood and tied ropes to record things. In 1957, the Chinese Communist Party and government helped the Hani create a script based on the Latin alphabet, which was put into practice in Honghe Prefecture and is still in use today. Honghe Prefecture is located between 101°47′ to 104°16′E and 22°26′ to 24°45′N and has a total area of 32 930 km^2^. It has four county-level cities, six counties and three autonomous counties and is located in southeast Yunnan Province. This prefecture connects Kunming and Qujing in the north, Wenshan in the east, Yuxi and Pu’er in the west and the Socialist Republic of Vietnam in the south and is crossed by the Tropic of Cancer. Honghe Prefecture is a multiethnic minority autonomous prefecture in the border area, where 10 ethnic groups have lived for a long time; the main groups are the Yi and Hani, of which the Hani population is 808 600. Thus, the Hani minority group in Honghe is of great interest to population genetics and anthropological investigations.

Unfortunately, there is no relevant research or reports on X-STRs in the Hani population in Yunnan Province. It is of great practical significance to explore the genetic characteristics of the Hani popu­lation and provide basic data for forensic practice. To establish a database of X-STRs of the Hani ethnic minority population of Yunnan Province in China, we investigated the genetic polymorphisms of 16 X-STR loci in the Hani group from Yunnan Province and compared pairwise genetic distances with the other previously published populations.

## Materials and methods

### Sample preparation and DNA extraction

A total of 451 peripheral blood samples were collected from unrelated heathy Hani individuals (116 males and 335 females) living in Honghe Hani and Yi Autonomous Prefecture in Yunnan Province in China after obtaining written informed consent. The study was approved by the Ethics Committee of Kunming Medicine University. Genomic DNA was extracted using the Chelex-100 method [12].

### PCR amplification and X-STR genotyping

The extracted DNA was amplified using the Multiplex PCR System Goldeneye 17X Kit (Peoplespot Incorporation, Beijing, China; including 16 X-STR loci, namely, DX6795, DXS9902, DXS8378, HPRTB, GATA165B12, DXS7132, DXS7424, DXS6807, DXS6803, GATA172D05, DXS6800, DXS10134, GATA31E08, DXS10159, DXS6789, DXS6810) on a GeneAmp 9700 PCR System (Applied Biosystems, Carlsbad, CA, USA) according to the manufacturer’s instructions. We also amplified 9948 control DNA and H_2_O as the positive and negative controls, respectively. Capillary electrophoresis of the amplified products was performed using an ABI 3130XL Genetic Analyzer (Applied Biosystems), and the results were subsequently analyzed using GeneMapper ID v3.2 software (Applied Biosystems).

### Statistical analyses

The allele frequencies were calculated using Modified-powerstate (Promega, Madison, WI, USA) [13]. Linkage disequilibrium (LD) among males between all pairs of loci was calculated using Arlequin Version 3.5.2.2 [14]. Hardy–Weinberg equilibrium (HWE) of females was estimated using the same software. General forensic parameters, such as polymorphism information content (PIC), power of discrimination in females (PD_F_) and males (PD_M_), mean paternity exclusion chance for ChrX markers in trios involving daughters (MEC trio), mean paternity exclusion chance for ChrX (MEC duo), power of exclusion (PE) and expected heterozygosity (Hexp), were estimated using the online calculation tool associated with the ChrX-STR.org 2.0 database (http://xdb.qualitype.de/xdb/index.jsf) [15]. Nei’s genetic distances were evaluated using Phylip 3.695 [16]. Based on the Nei’s genetic distances, a principal component analysis (PCA) was performed using Multivariate Statistical Package (MVSP) version 3.22 software [17], a multidimensional scaling (MDS) plot was constructed by SPSS Version 19.0 (IBM, Armonk, NY, USA) and a neighbouring-joining (N-J) phylogenetic tree was constructed in Mega v7.0 [18].

## Results and discussion

### Allele frequencies and forensic parameters

No significant differences in allele frequency distribution were observed between male and female samples by Fisher’s exact test after Bonferroni correction (*P* = 0.05/16; Supplementary Table S1). Therefore, both samples were pooled together and the allele frequencies and forensically relevant parameters were calculated and are shown in Supplementary Table S2. A total of 129 alleles were identified, with the associated allele frequencies spanning from 0.0018 to 0.7601 among our investigated individuals. A rare allele, 23.2, was detected at the DXS10159 locus and three rare alleles, 34.2, 39.3 and 44.3, were detected at DXS10134. The number of alleles at each locus varied from 5 at DXS8378 and DXS6810 to 22 at DXS10134. The PIC ranged from 0.3796 to 0.8855, and DXS10134 and DXS6800 were considered to be the most polymorphic (PIC = 0.8855; 22 alleles) and least polymorphic (PIC = 0.3796; 6 alleles), respectively. These results are consistent with previous studies in which the DXS10134 locus was shown to be highly polymorphic [8]. However, most of the X-STR kits on the market have not added the DXS6800 locus. This locus had a low PIC in this study; thus, a locus with higher polymorphisms should be used in future multiplex amplifications. The Hexp ranged from 0.4025 to 0.8943, the PE ranged from 0.1154 to 0.7838, the PD_M_ ranged from 0.4025 to 0.8943 and the PD_F_ ranged from 0.6201 to 0.9801. The combined PD_M_ and PD_F_ were 0.999 999 998 433 993 and 0.999 999 999 999 998, respectively.

### LD analyses

Of the 16 X-STR loci reported here, five loci (DXS6807, DXS8378, DXS9902, DXS6795 and DXS6810) are on the short arm, two loci are on the centromere (DXS10159 and DXS7132), and nine loci (DXS6800, DXS6803, DXS6789, DXS7424, GATA172D05, GATA165B12, HPRTB, GATA31E08 and DXS10134) are on the long arm of the X chromosome.

No significant deviations from HWE were observed in the female subsample (Supplementary Table S3). The results of the LD analysis for pairs of X-STR loci showed no significant LD after Bonferroni’s correction for multiple testing (*P* = 0.05/120; Supplementary Table S4).

### Interpopulation comparisons

The allele frequencies of X-STR loci in the Yunnan Hani population were compared to the available data of other groups, including Yunnan Bai [19], Xinjiang Kazakh [20] and Uyghur [21], Sichuan Yi [22], Guangxi Zhuang and Mulao [23], Zhejiang She [24], Guizhou Bouyi [25] and Miao [26], Guizhou Sui [27], Sichuan [28] and Qinghai Tibetan [29], Xinjiang [30] and Inner Mongolia Mongolian [29], Shanghai Han [31], Hunan Han [32], Sichuan Han [33] and Guizhou Han [34] based on the allele frequencies of seven overlapping X-STR loci (DXS8378, HPRTB, DXS7132, DXS7424, DXS10134, DXS10159 and DXS6789) .

**Figure 1.**
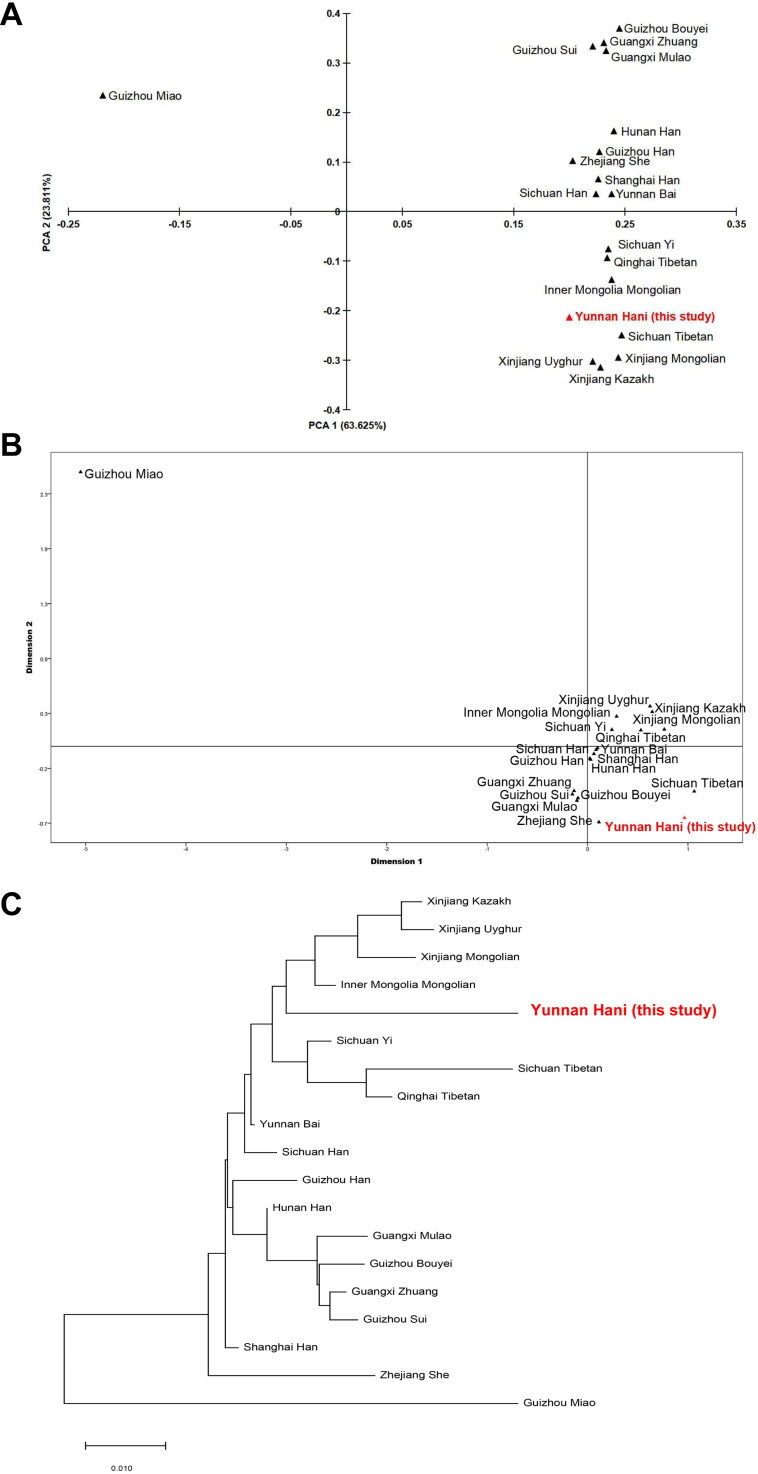
Genetic structure and population relationship between Yunnan Hani and 18 Chinese populations. (A) principal component analysis (PCA) revealed the genetic relationship based on the first two components; (B) multidimensional scaling (MDS) plots showed the population relationship based on Nei’s genetic distances; (C) neighbour-joining tree revealed the phylogenetic relationship of the 19 Chinese populations.

We calculated Nei’s genetic distance based on allele frequency and listed it in Supplementary Table S5. Among the results, Guizhou Miao and Sichuan Tibetan had the largest genetic distance (0.11891), followed by Guizhou Miao and Yunnan Hani (0.11468), and Yunnan Bai and Shanghai Han had the smallest genetic distance. To elucidate the genetic relationship and background among 19 ethnic groups, we performed a PCA study. The first 10 PCAs can extract a total of 99.826% of the genetic variations, and the first two principal components accounted for 87.436% of the total variance (PCA1 and PCA2 are responsible for 63.625% and 23.811%, respectively) from the 19 populations. In the PCA dimensional plots constructed on the basis of PCA1 and PCA2, Yunnan Hani is clustered in the same quadrant with the Sichuan Tibetan, Inner Mongolia Mongolian, Xinjiang Mongolian, Xinjiang Uyghur, Xinjiang Kazakh, Qinghai Tibetan and Sichuan Yi. The Guizhou Miao is obviously separated from all ethnic groups in one quadrant, while other ethnic groups are in the same quadrant. For further validation, we subsequently drew the MDS and N-J tree based on Nei’s genetic distance matrix, and the results are consistent (Figure 1, Supplementary Table S5).

Our results show that the evolutionary relationship of the Hani is relatively independent and relatively close to the Yi, Tibetan, Kazakh, Uyghur and Mongolian populations. This finding may be because the Tibetan, Yi and Hani populations belong to the ancient Qiang nationality and the Sino-Tibetan language family [27, 34] and they are similar in genetic background. The three ethnic groups in Xinjiang are obviously clustered into one group, and previous studies have found similar results [27, 33, 34], which may be due to their close geographical location and membership in the Altaic language family, which resulted in more intermarriage and more gene exchange. The Bai nationality in Yunnan Province has a close relationship with most Han populations, which has been found in previous studies [35], and this finding may be related to both Bai and Han belonging to the ancient Qiang nationa­lity. The Bai nationality has absorbed a large number of Han populations and cultures in its formation and development. Due to gene exchange with the Han nationality, the Bai is closer to the Han nationality [35]. Bouyi and Sui in Guizhou are closely related to Mulao and Sui in Guangxi, which is consistent with the study of Yang et al. [36], which may be due to the fact that Buyi, Mulao, Zhuang and Sui belong to the ancient Baipu family. Among all the nationalities included in the analysis, the Miao people had the earliest divergence. In the phylogenetic tree, neighbouring populations and different nationalities in the same area appeared to have a closer evolutionary relationship except for the Guizhou Miao populations. These observations are consistent with the cultural and geographi­cal background and development of the populations.

In this study, 16 X-STR loci of 451 unrelated individuals of Hani nationality in Yunnan Province were genotyped and analyzed using the Goldeneye 17X Kit. All these loci are highly polymorphic in the Hani population in Yunnan Province except for DXS6800 and can be used for forensic, anthropological and human medical genetics research. The genetic distance between different ethnic groups was calculated, which is helpful for estimating the genetic relationships among ethnic groups. In summary, our study provides the first batch of X chromosome genetic polymorphism data of the Hani population in Yunnan Province of Southwest China, and the findings enrich the reference database of the Chinese minority population.

## Authors’ contributions

Liping Hu, Shengjie Nie and Xiufeng Zhang carried out the study conception and design. Jinyong Yao, Yangzhi Huang, Lei Gao, Jiameng Dai and Xiaokun Yuan performed sample collection and material preparation. Linlin Liu, Xiufeng Zhang and Liping Hu carried out data acquisition and analysis. Linlin Liu and Liping Hu drafted the manuscript. Liping Hu and Shengjie Nie performed manuscript review. All authors read and approved the final manuscript.

## Supplementary Material

Supplemental Material
