# Supplementary material for: Genetic polymorphisms of 16 X-STR loci in the Hani population from Southwest China
Source: Forensic Sci Res. 2021 Jun 16;7(2):196–201. doi: 10.1080/20961790.2021.1877389 (PMC9246014; doi:10.1080/20961790.2021.1877389)
Supplement: Supplemental Material [file TFSR_A_1877389_SM6160.zip › Supplementary tables of 1877389/Table S1.docx]

| **Locus** | **p-value** |
| --- | --- |
| DXS6795 | 0.8751 |
| DXS9902 | 0.8774 |
| DXS8378 | 0.2229 |
| HPRTB | 0.5639 |
| GATA165B12 | 0.4208 |
| DXS7132 | 0.7304 |
| DXS7424 | 0.1064 |
| DXS6807 | 0.0147 |
| DXS6803 | 0.2935 |
| GATA172D05 | 0.0926 |
| DXS6800 | 0.3472 |
| DXS10134 | 0.1877 |
| GATA31E08 | 0.2169 |
| DXS10159 | 0.418 |
| DXS6789 | 0.6399 |
| DXS6810 | 0.4898 |

**Table S1. Fisher's exact test for population differentiation per locus between allele frequencies of male and female samples**
